# Spatially resolved analysis of plutonium isotopic signatures in environmental particle samples by laser ablation-MC-ICP-MS

**DOI:** 10.1007/s00216-015-8876-y

**Published:** 2015-07-14

**Authors:** Stefanie Konegger-Kappel, Thomas Prohaska

**Affiliations:** Department of Chemistry, Division of Analytical Chemistry, Research Group Analytical Ecogeochemistry, University of Natural Resources and Life Sciences, Vienna, Konrad-Lorenz-Straße 24, 3430 Tulln, Austria; Office of Safeguards Analytical Services, Department of Safeguards, International Atomic Energy Agency (IAEA), Vienna International Centre, PO Box 100, 1400 Vienna, Austria

**Keywords:** Plutonium isotope ratios, Environmental contamination, Chernobyl nuclear power plant, (MC)-ICP-MS, Laser ablation

## Abstract

Laser ablation–multi-collector–inductively coupled plasma mass spectrometry (LA-MC-ICP-MS) was optimized and investigated with respect to its performance for determining spatially resolved Pu isotopic signatures within radioactive fuel particle clusters. Fuel particles had been emitted from the Chernobyl nuclear power plant (ChNPP) where the 1986 accident occurred and were deposited in the surrounding soil, where weathering processes caused their transformation into radioactive clusters, so-called micro-samples. The size of the investigated micro-samples, which showed surface alpha activities below 40 mBq, ranged from about 200 to 1000 μm. Direct single static point ablations allowed to identify variations of Pu isotopic signatures not only between distinct fuel particle clusters but also within individual clusters. The resolution was limited to 100 to 120 μm as a result of the applied laser ablation spot sizes and the resolving power of the nuclear track radiography methodology that was applied for particle pre-selection. The determined ^242^Pu/^239^Pu and ^240^Pu/^239^Pu isotope ratios showed a variation from low to high Pu isotope ratios, ranging from 0.007(2) to 0.047(8) for ^242^Pu/^239^Pu and from 0.183(13) to 0.577(40) for ^240^Pu/^239^Pu. In contrast to other studies, the applied methodology allowed for the first time to display the Pu isotopic distribution in the Chernobyl fallout, which reflects the differences in the spent fuel composition over the reactor core. The measured Pu isotopic signatures are in good agreement with the expected Pu isotopic composition distribution that is typical for a RBMK-1000 reactor, indicating that the analyzed samples are originating from the ill-fated Chernobyl reactor. The average Pu isotope ratios [^240^Pu/^239^Pu = 0.388(86), ^242^Pu/^239^Pu = 0.028(11)] that were calculated from all investigated samples (*n* = 48) correspond well to previously published results of Pu analyses in contaminated samples from the vicinity of the Chernobyl NPP [e.g. ^240^Pu/^239^Pu = 0.394(2) and ^242^Pu/^239^Pu = 0.027(1); Nunnemann et al. (J Alloys Compd 271–273:45–48, 1998)].

**Graphical Abstract:**
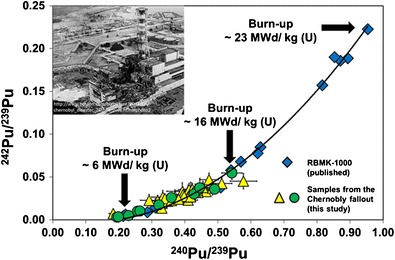
Evaluation of LA-MC-ICP-MS for spatially resolving ultra-trace Pu isotopic signatures stored in environmental radioactive particles, providing new insights into the Pu isotopic distribution of the Chernobyl reactor core at the time of the accident

Evaluation of LA-MC-ICP-MS for spatially resolving ultra-trace Pu isotopic signatures stored in environmental radioactive particles, providing new insights into the Pu isotopic distribution of the Chernobyl reactor core at the time of the accident

## Introduction

Monitoring of environmental contaminations with actinides is of special interest not only for the assessment of human hazards [1, 2] and timely radiation protection measures but also for gaining information about radiological impacts on flora and fauna [3]. Moreover, the knowledge of chemical and isotopic compositions of nuclear material, reflecting the origin, intended use and ongoing nuclear activities, is of particular importance for nuclear forensics [4] and nuclear safeguards [5].

Nowadays, radionuclides are mainly released into the environment due to nuclear fuel cycle operations, including releases from reprocessing plants or waste deposits and due to nuclear accidents [6–10]. However, in the twentieth century, large amounts of Pu were released into the environment as a consequence of stratospheric nuclear weapon tests that took place between 1945 and 1975, with a maximum emission around 1963 [11, 12]. The Pu isotopic signatures of this global fallout are well known today due to the establishment of regional Pu isotopic baselines [13], which helps to recognize additional Pu inputs into the ecosystem. The mean ^239^Pu concentration for surface soil is about 10^−13^ g g^−1^. Pu concentrations larger than 10^−12^ g g^−1^ are regarded as hazardous when accumulated in the human body [2].

About 1–2 % of the global Pu content present in the environment back in the 1980s [2] was emitted during the accident at the fourth unit of the Chernobyl nuclear power plant (Ch-NPP-4) on the 26th of April 1986. Pu was mainly released in form of fuel particles as it was associated with the uranium fuel that was emitted from the reactor core. It got deposited in the surrounding soil, mainly contaminating the 30-km zone around the accidental reactor [14, 15]. The deposited fuel particles varied, according to the scenarios during the accident, in their chemical compositions, morphologies and oxidation states [16]. The fate of fuel particles in the environment depends on both their chemical properties and environmental conditions [15]. Non-oxidized particles are regarded as relatively chemically stable, whereas the oxidized fraction is more susceptible to weathering [17] and dissolution in soil, which promotes the mobilization of radionuclides into the ecosystem [15]. Oxidation and weathering processes can furthermore lead to superficial cracking of particle surfaces and their transformation into radioactive clusters—so-called micro-samples—with sizes of up to several hundreds of micrometers [18].

A very well-established technique for analyzing Pu isotopes and other alpha-emitting radionuclides with half-lives less than 1000 years is alpha-spectrometry [19–21]. However, in the case of low activities and concentrations, as is mostly the case for environmental samples, measurement times of several days or even weeks might be required for gaining reliable analytical results. Moreover, only a sum activity of ^239^Pu and ^240^Pu can be obtained due to the similar alpha energies of these two isotopes (5.16 MeV for ^239^Pu and 5.17 MeV for ^240^Pu). Complimentary methods for Pu isotopic analysis can be found in the field of mass spectrometry (i.e. inductively coupled plasma mass spectrometry (ICP-MS) [12, 22], thermal ionization mass spectrometry (TIMS) [23], resonance ionization mass spectrometry (RIMS) [24, 25] and accelerator mass spectrometry (AMS) [19, 21]), which relevance is also highlighted by numerous reviews [12, 22, 26–30] dedicated to this topic. Especially analyses performed by ICP-MS are offering easy sample preparation, relatively low analysis costs and high sample throughput due to short analysis times, with all being merits for environmental sample analyses. In the last years, the combination of ICP-MS with laser ablation for direct solid sampling has also more and more found its way into the field of nuclear safeguards and nuclear forensics (e.g. [31–33]). While laser ablation allows to resolve a sample’s inherent isotopic and elemental information as well as within-sample inhomogeneities, ICP-MS offers high sensitivity for the analysis of very low actinide amounts [30, 34]. Because of this and its comparatively low susceptibility for molecular clusters at actinide mass-to-charge ratios, laser ablation (LA)-ICP-MS is regarded as an attractive complementary method to well-established mass spectrometric techniques (i.e. fission track–TIMS [35] and secondary ion mass spectrometry (SIMS)) for actinide particle analyses [32]. The attractiveness of LA-ICP-MS does not at last result from the circumstance that one could proceed very quickly from sample to sample in emergency situations, allowing a fast assessment of environmental contaminations and their origins. However, as LA-ICP-MS is a comparatively new method in this field, an expanded knowledge about its performance is required for further evaluating its applicability for various particle sample matrices. In a previous study that was published by Boulyga and Prohaska [18], the potential of LA-ICP-MS was demonstrated for the spatially resolved analysis of U, Nd and Ru/(Ru + Tc) isotope ratios in Chernobyl micro-samples.

The aim of this study was the adaptation and optimization of the LA-multi-collector (MC)-ICP-MS methodology for spatially resolved Pu isotope ratio measurements in radioactive fuel particle cluster samples that were collected in the same batch as those analyzed in the previous study [18]. A modified multi-collector set-up allowed to measure ultra-trace Pu isotope amounts, whereas the use of laser ablation sampling allowed to determining variations of the Pu isotopic composition both between and within individual fuel particle cluster samples. Up to now, only a very limited amount of data has been available for individual hot particle samples from the Chernobyl fallout [36, 37]. Since, in this study, a large number of individual particle cluster samples (*n* = 48) were analyzed and spatially resolved for the Pu isotopic composition, important insights are provided for the first time about the distribution of the spent fuel composition over the reactor core at the time of the accident.

## Experimental

### Samples and sample preparation

Micro-samples (i.e. radioactive fuel particle clusters embedded in a soil matrix) emitted during the Chernobyl accident were sampled in the vicinity of the Chernobyl nuclear power plant (Ch-NPP) (8 km to the north-west of the Ch-NPP) in 1992. The soil samples were taken in an area that has remained anthropogenically untouched since the accident. Sampling, sample preparation as well as nuclear track radiography analyses for particle identification and localization were performed at the Institute of Power Engineering, Minsk, Belarus, in 1992. Experimental details are given elsewhere [18]. The identified micro-samples, which were measured by nuclear track radiography, yielded sizes and surface alpha activities from about 200 to 1000 μm and from 3 to 38 mBq, respectively. The micro-samples were embedded in cellulose acetate membrane filters (OE 67, Whatman; GE Healthcare, UK). Exposure to acetone vapour resulted in transparent membranes, which were affixed to circular glass plates (*d* = 25 mm) by means of commercially available glue [38]. The glass plates were directly put into the laser ablation cell.

### Reagents and certified reference material

The analyses of the Chernobyl micro-samples included measurements of 1 % (*m*/*m*) nitric acid (HNO_3_) as blank solution and CRM U500 (New Brunswick Laboratory, U.S. Department of Energy, USA), a U solution isotopic reference material, for instrument optimization and the determination of external correction factors for correcting mass bias and secondary electron multiplier gain. Sixty-five percent (*m*/*m*) HNO_3_ (analytical reagent grade; Merck KGaA, Darmstadt, Germany) was diluted with reagent grade type I water (18.2 MΩ/cm at 25 °C, Ultra Clear Basic Reinstwassersystem; SG Wasseraufbereitung und Regenerierstation GmbH, Barsbüttel, Germany). Both were purified by sub-boiling distillation (Milestone-MLS GmbH, Leutkirch, Germany) prior to use.

### LA-MC-ICP-MS instrumentation

Pu isotope ratio measurements were accomplished with a double-focusing high-resolution sector field MC-ICP-MS (Nu Plasma HR; Nu Instruments Limited, Wrexham, UK) that was coupled to a solid-state nanosecond LA system (UP193 Solid State Laser Ablation System (Nd:YAG); ESI–NWR Division, Electro Scientific Industries, Inc., Portland, CA, USA) for direct solid sample introduction. A membrane desolvation system (DSN-100; Nu Instruments Limited, Wrexham, UK) was connected in parallel to the laser ablation system for measuring a U solution isotopic reference material (CRM U500) for determining external correction factors (mass bias and secondary electron multiplier gain) in between the analyses of the solid micro-samples according to a standard–sample bracketing approach. The desolvation unit allowed introducing a dry aerosol into the ICP, thus reducing interferences by molecular ions, such as oxides and hydrides [14, 28]. No solution was aspirated during laser ablation.

**Fig. 1:**
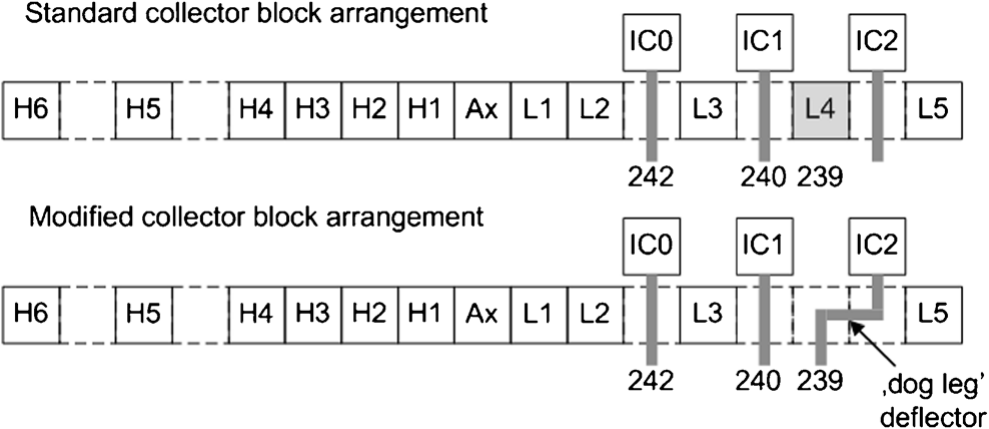
Collector block arrangements of the Nu Plasma HR MC-ICP-MS (*H* Faraday cup high mass side, *Ax* Faraday cup axial mass, *L* Faraday cup low mass side, *IC* secondary electron multiplier)

Based on the results of a previous study [18], the standard collector block of the multi-collector was modified for simultaneously measuring ultra-trace ^239^Pu^+^, ^240^Pu^+^ and ^242^Pu^+^ isotope amounts with three secondary electron multipliers (SEMs) (see Fig. 1). A deflection lens deflected the ion beam into the low mass side SEM for measuring the above-mentioned isotopes (see “Results and discussion” section for more details). Instrumental parameters are summarized in Table 1.

**Table 1:** LA-MC-ICP-MS instrumental parameters

Laser (New wave ‘UP 193’)	
Ablation mode	Static point ablation
Wavelength, nm	193
Pulse length, ns	3
Energy density, J cm^−2^	1.70–5.83
Power density, GW cm^−2^	0.66–2.06
Repetition rate, Hz	10
Spot size, μm	100–120
Ar carrier gas flow rate, L min^−1^	0.7
Liquid sample introduction	
System type	DSN-100
Nebulizer	PFA 100
Sample uptake rate, μL min^−1^	130
Nebulizer gas pressure, Pa	∼2 × 10^5^
Hot gas flow rate, L min^−1^	∼0.25
Membrane gas flow rate, L min^−1^	∼1.4
Spray chamber temperature, °C	112–116
Membrane temperature, °C	119–123
MC-ICP-MS (Nu Plasma HR)	
RF power, W	1300
Auxiliary gas flow rate, L min^−1^	0.95
Cool gas flow rate, L min^−1^	13
Mass separation	1
Isotopes monitored	^235^U, ^236^U, ^238^U, ^239^Pu, ^240^Pu, ^242^Pu
Resolution, m/∆m	300 (low resolution)
Detection system	IC0^a^, IC1^a^, IC2^a^
High voltages, source and transfer lens parameters	Optimized for optimal sensitivity
Voltages applied to collector and multipliers	Optimized for optimal peak shape and alignment
Data acquisition mode	TRA (acquisition time per data point = 1 s)

*TRA* time-resolved analysis

^a^Secondary electron multiplier

### Data Processing

The measured ^239^Pu^+^, ^240^Pu^+^ and ^242^Pu^+^ signal intensities were recorded in time-resolved analysis mode with an acquisition time of 1 s per data point. A typical laser ablation analysis performing static point ablation lasted for about 20–40 s, excluding the time for measuring Ar gas blanks. Ar gas blanks were recorded for about 10 s prior to the start of the ablation of the micro-samples in order to guarantee a complete wash-out of the previous ablation. The maximum recorded signal intensities per ablation ranged from about 160–600,000 counts per second (cps) for ^239^Pu, 60–284,000 cps for ^240^Pu and 7–19,000 cps for ^242^Pu. Dead time correction of the measured intensities was automatically performed in the Nu Plasma software, applying dead times of 8.5, 10 and 11 ns for SEMs IC0, IC1 and IC2, respectively.

Calculation of the ^242^Pu/^239^Pu and ^240^Pu/^239^Pu isotope ratios was performed with the help of the slope of scatter plot regression lines [33, 39]. The main advantage of this data processing strategy is that each data point, including the blank, is taken into account and that the contribution of each data point to the linear fit depends on its signal intensity. Thus, higher signal intensities, usually yielding more precise data than lower signal intensities, are dominating the fit, which is regarded to be advantageous, especially when processing time-resolved ablation profiles [33]. The ^242^Pu/^239^Pu and ^240^Pu/^239^Pu isotope ratios were corrected for mass bias and secondary electron multiplier gain. The external correction factors were derived by measuring ^238^U/^235^U and ^236^U/^235^U isotope ratios in CRM U500 for at least 10 min in a bracketing approach, assuming similar mass bias effects of U and Pu [i.e. f(^236^U/^235^U) = f(^240^Pu/^239^Pu) and f(^238^U/^235^U) = f(^242^Pu/^239^Pu)]. ^238^U and ^242^Pu were measured with IC0, ^236^U and ^240^Pu with IC1, and ^235^U and ^239^Pu with IC2. The external correction factors for correcting the ^240^Pu/^239^Pu and ^242^Pu/^239^Pu isotope ratios were calculated by dividing the certified ^238^U/^235^U and ^236^U/^235^U isotope ratios with the U isotope ratios measured in CRM U500. ^238^U was measured with IC0, whereas ^236^U and ^235^U were determined with IC1 and IC2, respectively. One percent (*m*/*m*) HNO_3_ was analyzed for blank correction. Both blank and CRM U500 signal intensities of the isotopes of interest were assessed by calculating an average of six blocks, with each block representing an average of 100 data points.

In the case of homogenous isotope ratio distributions within one micro-sample, ^242^Pu/^239^Pu and ^240^Pu/^239^Pu isotope ratios of more than two single static point ablations (on one micro-sample) were pooled together by calculating the weighted mean of replicate LA measurements. Weighting factors were derived from the theoretical precision of the measured signal intensities.

### Uncertainty assessment

Computation of expanded (*k* = 2) measurement uncertainties (*U*) was accomplished with the GUM Workbench Pro software (version 2.4; Metrodata GmbH, Weil am Rhein, Germany) according to ISO/GUM [40] and Eurachem [41] guidelines. Single parameters that were propagated for the expanded combined standard measurement uncertainties included blanks (1 % (*m*/*m*) HNO_3_ for liquid and dark noise for LA measurements), dead times, SEM gains, measurement repeatabilities, uncertainties of the certified ^238^U/^235^U and ^236^U/^235^U isotope ratios measured in CRM U500, ^238^U^+^ peak tailing at the mass *m* − 2*u* and contributions from ^235^U^1^H hydride ions at *m* = 236*u* (CRM U500 measurements only) and ^238^U^+^ peak tailing at *m* + 1*u*, *m* + 2*u* and *m* + 4*u* (Pu isotope ratio measurements only). The within-measurement repeatability of laser ablation analyses was calculated from the uncertainty of linear regression slopes, whereas the within-measurement repeatability of liquid measurements was calculated from the standard deviation of six measurement blocks.

The ^238^U^+^ peak tailing at *m* − 2*u* did not significantly contribute to the expanded combined standard measurement uncertainties of the CRM U500 measurements. This can be explained by the fact that peak tailing is less pronounced for larger ratios as is the case in this study for ^236^U/^235^U (i.e. 0.0015192(31)). Since the ^242^Pu/^239^Pu and ^240^Pu/^239^Pu isotope ratios of the Chernobyl samples were in the same order of magnitude or larger, peak tailing of ^239^Pu into the masses of ^240^Pu and ^242^Pu was neglected in the uncertainty propagation of the results of the micro-sample measurements.

Peak tailing effects of ^238^U^+^ ions, originating from U present in the analyzed micro-sample cluster, on the masses of the Pu isotopes at *m* + 1*u*, *m* + 2*u* and *m* + 4*u* were assessed by measuring U500 at an axial mass of *m* = 245*u*. This procedure allowed assessing the peak tailing of ^238^U^+^ ions at the masses of *m* + 1*u*, *m* + 2*u* and *m* + 4*u*. The relative intensities at the masses *m* = 242*u*, *m* = 240*u* and *m* = 239*u* that were normalized to the intensity of ^238^U^+^ ions were about 4 × 10^−7^, 3 × 10^−6^ and 3 × 10^−5^. The relative intensities at the masses of *m* = 242*u* and *m* = 240*u* are reflecting the peak tailing from ^238^U^+^ ions, whereas the relative intensities at the mass *m* + 1*u* are representing both peak tailing and a contribution from ^238^U^1^H^+^ hydride ions.

## Results and discussion

### Spatially resolved Pu isotopic analysis of environmental particle samples

#### Micro-sample pre-selection

Generally, the size of the laser beam can be a major limitation for single particle analysis, especially for particles with sizes in the low or sub-micrometer range [33]. Hence, particle pre-identification, selection and separation from their matrix or from neighbouring particles are considered beneficial for decreasing the risk of simultaneously ablating neighbouring particles and obtaining mixed isotopic information [31]. In the case of the particle samples analyzed in this study, the identification and pre-selection of particles of interest from the collected soil was particularly deemed necessary for increasing the LA sample throughput.

Theoretically, pre-selection of particles or areas of interest could be accomplished by performing a fast pre-ablation of the sample surface. However, due to limited sample material, the applicability of such a pre-ablation is limited for particle samples as well as for samples containing ultra-trace actinide amounts as was the case in this study, where individual micro-samples contained Pu amounts in the picogram to femtogram range. In the case of the Chernobyl micro-samples, relatively large spot sizes (100–120 μm) and laser repetition rates (10 Hz) were required for obtaining a good, measurable signal for improving accuracy and precision. In the case of a pre-ablation that yielded useful data for pre-selection, not enough sample material would have been left for the actual isotope ratio analysis. Therefore, combining a pre-selection method like nuclear track radiography and LA-MC-ICP-MS for environmental particle analysis, as performed in this study, has been regarded as beneficial for decreasing the LA-ICP-MS analysis time and thus increasing sample throughput. As a result of the applied laser ablation spot sizes and the resolving power of nuclear track radiography, the resolution for identifying individual particles was limited to 100–120 μm in this study.

#### MC-ICP-MS collector block modification

In contrast to other studies (e.g. [42–44]), where ultra-trace concentration levels of Pu isotopes in environmental samples had been determined by applying a single collector ICP-MS, a multi-collector-ICP-MS instrument was applied here, especially following the need that the isotope ratio has to be determined on short transient signals to not compromise measurement precision and accuracy of Pu isotope ratio measurements. Similar to a previous study on U, Nd and Ru/(Ru + Tc) isotope ratios [18], the standard collector block of the Nu Plasma HR multi-collector was modified for measuring ^239^Pu^+^, ^240^Pu^+^ and ^242^Pu^+^ simultaneously with three SEMs (Fig. 1). The standard collector block arrangement was modified using a so-called ‘dog leg’ deflector lens that was inserted at the L4 Faraday cup position and allowed to guide the ^239^Pu^+^ ion beam from the L4 position into IC2. This set-up was different from the previous study [18], where the ion pathway was redirected at the high mass side for measuring all isotopes with SEMs. The modification that is shown in Fig. 1 was deemed necessary for overcoming the lack of sensitivity of Faraday cups for measuring the very low Pu amounts (pg to fg amounts) present in the Chernobyl micro-samples.

### Pu isotopic signatures determined in the Chernobyl fallout

**Fig. 2:**
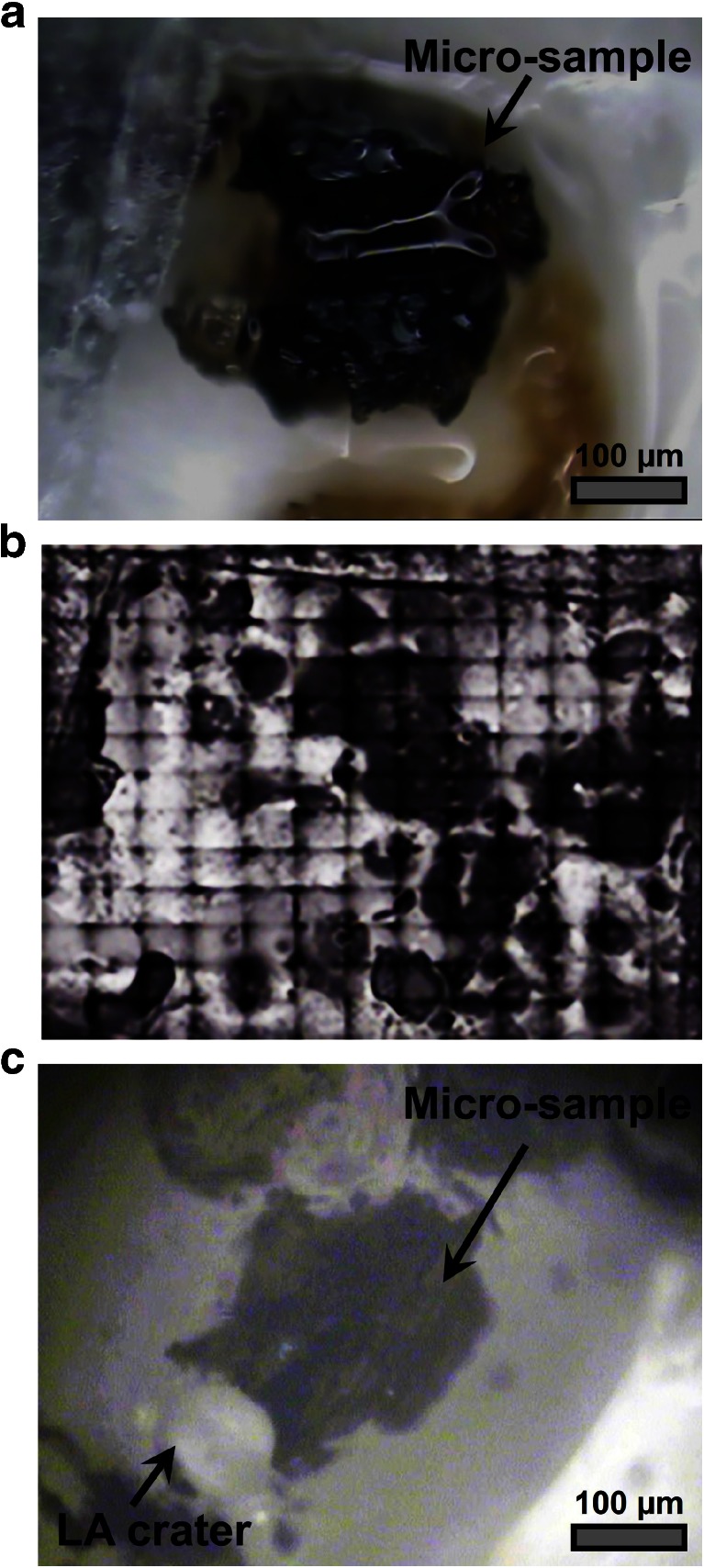
Depiction of micro-samples (radioactive fuel particle clusters) that were collected in the vicinity of the accidental Chernobyl reactor: (**A**) typical micro-sample embedded in a transparent membrane after pre-selection by nuclear track radiography, (**B**) LA sample map overview of a transparent membrane (∼507 mm^2^) containing several micro-samples (*dark spots*) and (**C**) typical micro-sample after performing one static point ablation

The micro-samples (Fig. 2) investigated in this study were regarded as ideal test material for extending the understanding about the performance of LA-MC-ICP-MS for spatially resolving radionuclide isotopic signatures in environmental particle samples as they exhibited low surface alpha activities (3–38 mBq) and ultra-trace Pu amount levels (pg to fg). Since the particles were emitted during the explosion of the accidental RBMK-1000 Chernobyl reactor, which typical characteristic was fuel replacement during operation, varying isotopic distributions of the micro-samples were presumed. Another advantage of these fuel particle cluster samples was the presence of isotopic heterogeneities within micro-samples, probably due to the generation of particle agglomerates during the explosion or of clusters due to weathering processes after their deposition [18]. This characteristic as well as the sizes of these cluster samples (200–1000 μm) allowed to investigate the resolving limits of the here-proposed analytical method.

#### Homogeneous Pu isotopic compositions

**Fig. 3:**
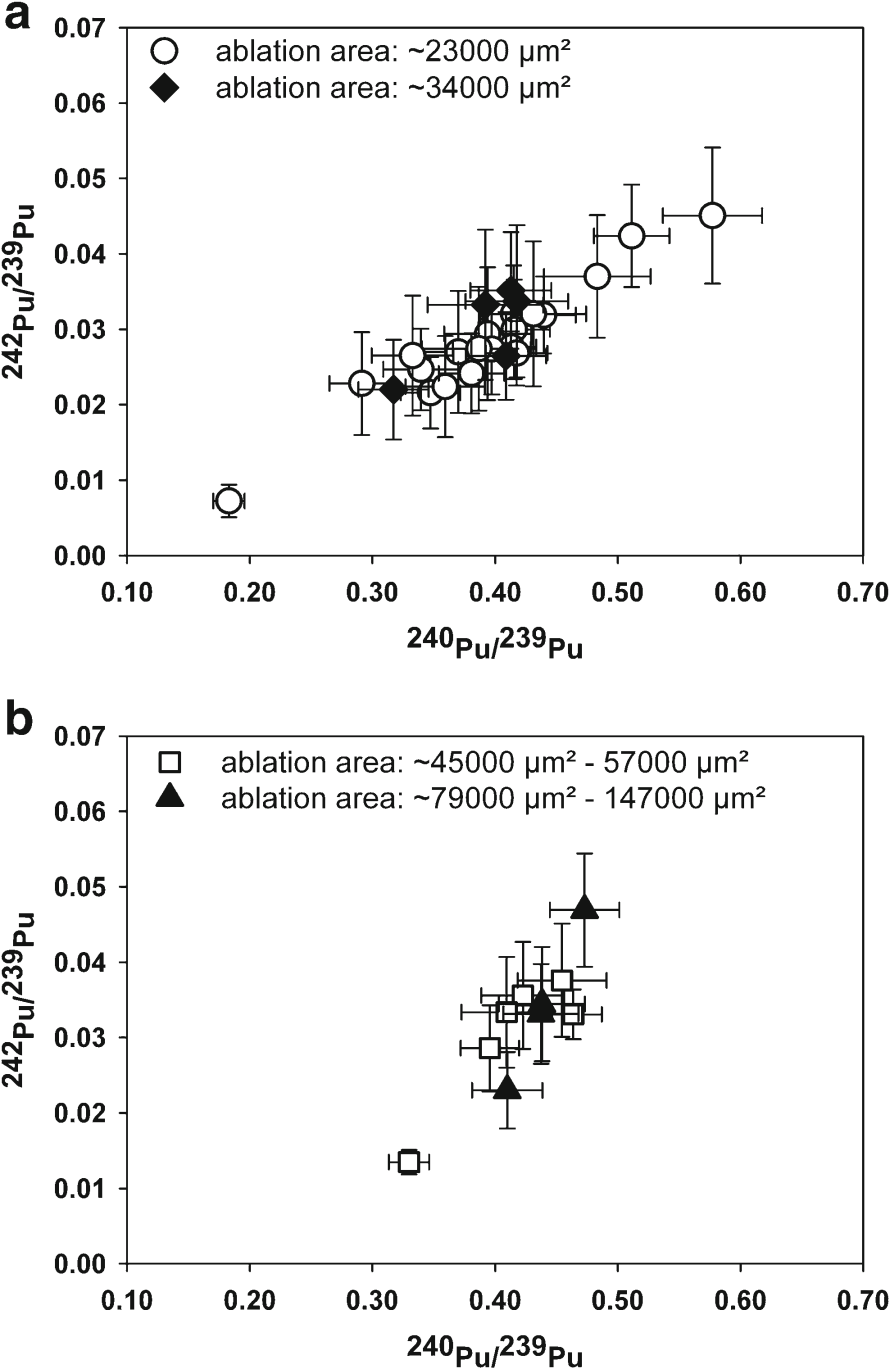
Pu isotopic compositions of micro-samples (*n* = 35) collected in the vicinity of the accidental ChNPP-4. ^242^Pu/^239^Pu and ^240^Pu/^239^Pu isotope ratios were calculated as weighted means of isotope ratios measured per micro-sample. The distribution of the Pu isotopic compositions is shown for smaller (**A**) and larger (**B**) micro-samples (sizes are expressed as ablation areas). Expanded (*k* = 2) measurement uncertainties (*U*) are displayed in form of *error bars*

Individual micro-samples were considered to be homogeneous if several static point ablations (*n* ≥ 2, spot size = 100 μm) on the same micro-sample yielded no significant differences for their Pu isotope ratios on a 95 % confidence interval (*k* = 2). In Fig. 3, the Pu isotopic compositions that were determined for micro-samples, which showed within-sample homogeneity but between-sample heterogeneity, are depicted. An estimate of the sizes of the micro-samples can be derived from the respective ablation areas, which ranged from approximately 0.023–0.034 mm^2^ for smaller clusters (Fig. 3A) and from 0.045 to 0.147 mm^2^ for larger ones (Fig. 3B).

**Fig. 4:**
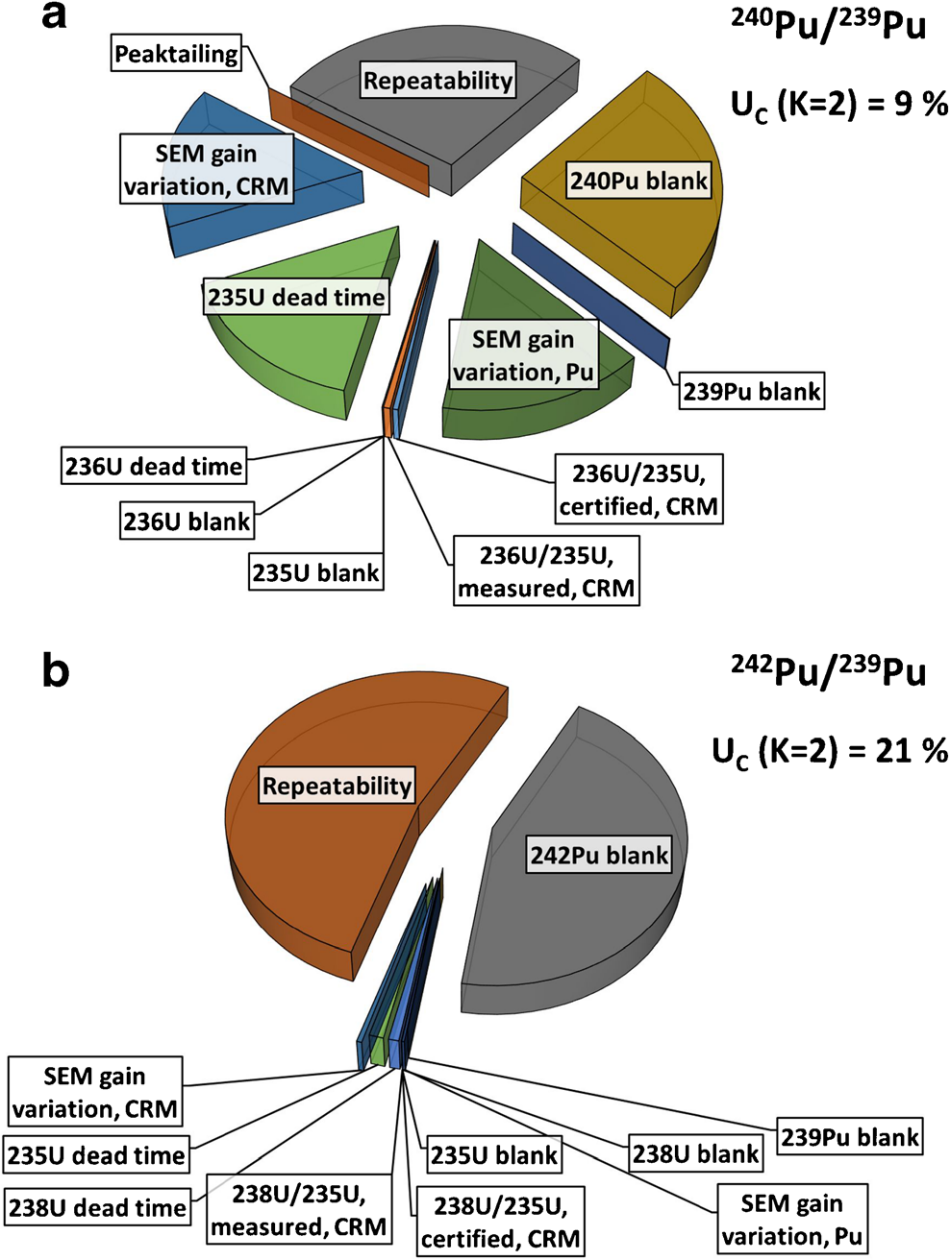
Parameters contributing to the expanded measurement uncertainties (*U*) (*k* = 2) of (**A**) ^240^Pu/^239^Pu and (**B**) ^242^Pu/^239^Pu

The isotopic compositions were calculated as weighted means of ^242^Pu/^239^Pu and ^240^Pu/^239^Pu, respectively, isotope ratios determined per micro-sample if the isotope ratios did not yield a significant difference within the expanded (*k* = 2) measurement uncertainties. The expanded uncertainties (*k* = 2) for individual spot ablations ranged from 4 to 46 % for ^242^Pu/^239^Pu and from 5 to 12 % for ^240^Pu/^239^Pu isotope ratios. Repeatability of LA measurements and the ^240^Pu blank were identified as main contributors (Fig. 4A) to the uncertainty for ^240^Pu/^239^Pu isotope ratio measurements. Other contributing parameters were SEM gain variations (both LA and liquid CRM measurements) and the dead time in case of the CRM measurement of the major isotope (^235^U). In case of ^242^Pu/^239^Pu isotope ratio measurements (see Fig. 4B), repeatability of the LA measurements and ^242^Pu blank were identified as main contributors, whereas blank contribution became less pronounced with ^242^Pu count rates higher than 100 cps. The within-LA measurement repeatabilities (internal precisions) for individual spot ablations ranged from 0.6 to 34.0 % and from 0.2 to 5.1 % for ^242^Pu/^239^Pu and ^240^Pu/^239^Pu, respectively. In comparison, external precisions (calculated as relative standard deviation of individual spot ablations per homogenous micro-sample) ranged from 0.3 to 37.1 % for ^242^Pu/^239^Pu and from 0.2 to 8.9 % for ^240^Pu/^239^Pu.

All micro-sample clusters that are depicted in Fig. 3 showed within-sample homogeneity for their Pu isotopic composition. As micro-samples are not only representing single fuel particles but also fuel particle agglomerates, it can be considered that these isotopically homogeneous micro-samples had probably been produced by fuel particles that originated from the same reactor fuel assemblies.

#### Heterogeneous Pu isotopic compositions

**Fig. 5:**
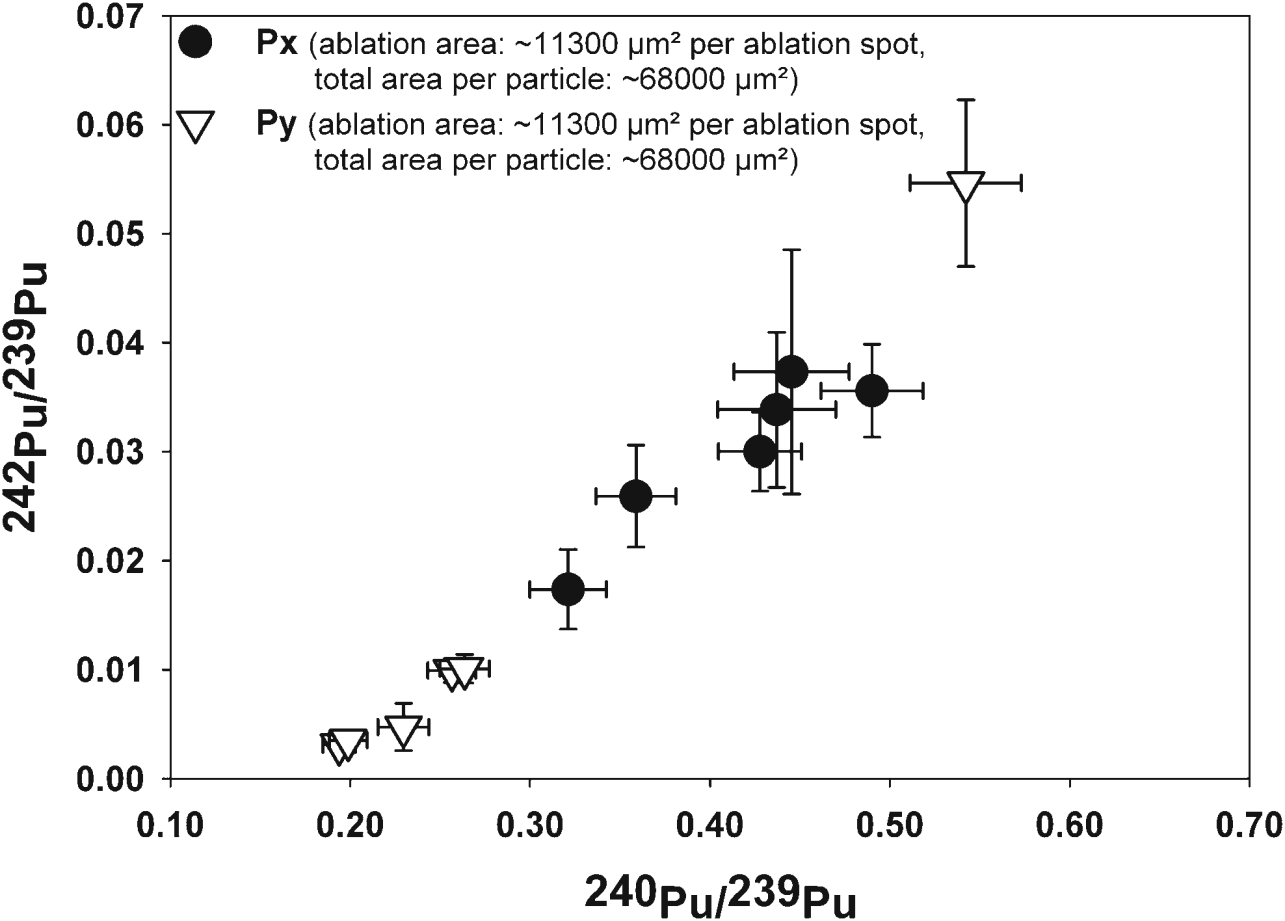
Heterogeneous distribution of ^242^Pu/^239^Pu and ^240^Pu/^239^Pu isotope ratios measured in two Chernobyl micro-samples, Px and Py. Expanded (*k* = 2) measurement uncertainties (*U*) are displayed the form of *error bars*

In case of two micro-samples, significant within-sample heterogeneities were identified for their Pu isotopic composition (Fig. 5). Each micro-sample was analyzed by six subsequent static point ablations with a spot size of 100 μm. It is believed that these two micro-samples were created by two or more fuel particles, which originated from fuel assemblies with different burn-up grades, so that the isotopic compositions of these micro-samples represent mixtures of several fuel particles with different burn-up grades.

The presence of varying Pu isotope ratios within individual micro-samples is also in good agreement with an investigation published by Boulyga and Prohaska [18], who observed heterogeneities in the U isotopic signatures of micro-samples that were collected in the same batch as those analyzed in this study. They assumed an encapsulation of particles with different U isotopic signatures in one micro-sample. It is still unclear if those particles got mixed during the explosion or during weathering and dissolution in soil. However, the presence of varying Pu or U isotopic signatures within one sample demonstrates the necessity of analytical methods that are capable of spatially resolving the inherent isotopic information.

#### Source attribution

A sample’s isotopic signature poses, like a human fingerprint, a distinct parameter, which can help to identify a sample’s origin, providing that information, with which those isotopic signatures can be compared, is available in the literature or from other reports. In the case of Pu inputs into the environment due to nuclear weapon tests and nuclear accidents, much effort has been made in the past for establishing global Pu isotopic baselines [13], whereupon the Chernobyl fallout has been of particular interest. An overview about published Pu isotope ratios that were determined in both soil and single radioactive particles is given in Table 2 for comparison. The average ^242^Pu/^239^Pu and ^240^Pu/^239^Pu isotope ratios (0.388(86) and 0.028(11) for ^240^Pu/^239^Pu and ^242^Pu/^239^Pu; calculated as average and 1 × SD) that were calculated from the results of all micro-samples (*n* = 48) measured in this study are in good agreement with previously published results from bulk analyses of soil samples. Overall, the analyzed micro-sample entity (*n* = 48) showed a variation from low to high Pu isotope ratios, ranging from 0.007(2) to 0.047(8) for ^242^Pu/^239^Pu and from 0.183(13) to 0.577(40) for ^240^Pu/^239^Pu. It should be noted that very limited data are provided in the literature regarding the Pu isotopic composition of individual particles collected in the vicinity of the accidental Chernobyl reactor [36, 37]. Thus, no information about the Pu isotopic distribution has been available so far for the Chernobyl fallout. The present study reports for the first time variations of Pu isotope ratios in the Chernobyl fallout, which were not assessed previously due to the limited analyses of individual particles or micro-samples.

**Table 2:** Published Pu isotope ratios of the Chernobyl fallout and the reactor core

Literature reference	^240^Pu/^239^Pu	^242^Pu/^239^Pu	Sample	Method
Muramatsu et al. [45]	0.403(9)^a^	n.a.	Soil (*n* = 8)	ICP-MS
Boulyga and Becker [43]	0.396(14)^a^	n.a.	Soil (*n* = 8)	ICP-MS
Nunnemann et al. [24]	0.394(2)^b^	0.027(1)^b^	Soil (*n* = 1)	RIMS
Erdmann et al. [25]	0.378(2)^a,b^	0.024(1)^a,b^	Soil	RIMS
Boulyga et al. [36]	0.329(16)^b^	0.021(3)^b^	Hot particle (*n* = 1)	RIMS
Wendt et al. [37]	0.378(2)^b^	0.088(1)^b^	Hot particle (*n* = 1)	RIMS
Kirchner and Noack [46]	0.56^a,c^	0.044^a,c^	Reactor core	Calculation
Begichev et al. [47]	0.39^a,c^	0.045^a,c^	Reactor core	Calculation
This study	0.388(86)^a^	0.028(11)^a^	Fuel particle clusters (*n* = 48)	LA-MC-ICP-MS
This study	0.183(13) to 0.577(40)	0.007(2) to 0.047(8)	Fuel particle clusters (*n* = 48)	LA-MC-ICP-MS

^a^Average values

^b^Isotope ratios were calculated from published isotopic compositions

^c^Isotope ratios were calculated from published activity data

The observed variations can be explained by the fact that spent fuel was gradually replaced with freshly enriched uranium in the Chernobyl RBMK-1000 reactor during its operation. That resulted in different irradiation histories, and thus, in varying burn-up grades of the fuel assemblies over the reactor core at the time of the accident [47]. The differences in the burn-up over the reactor core were reflected in different ^242^Pu/^239^Pu and ^240^Pu/^239^Pu isotope ratios in the fuel assemblies. While ^239^Pu is generated via neutron capture in ^238^U and subsequent β^−^ decay, ^240^Pu, ^241^Pu and ^242^Pu are generated via neutron capture (*n*,*γ*) in ^239^Pu. Hence, higher fuel burn-up led to an increase of the heavier Pu isotope fraction and as a consequence to higher ^242^Pu/^239^Pu and ^240^Pu/^239^Pu isotope ratios.

**Fig. 6:**
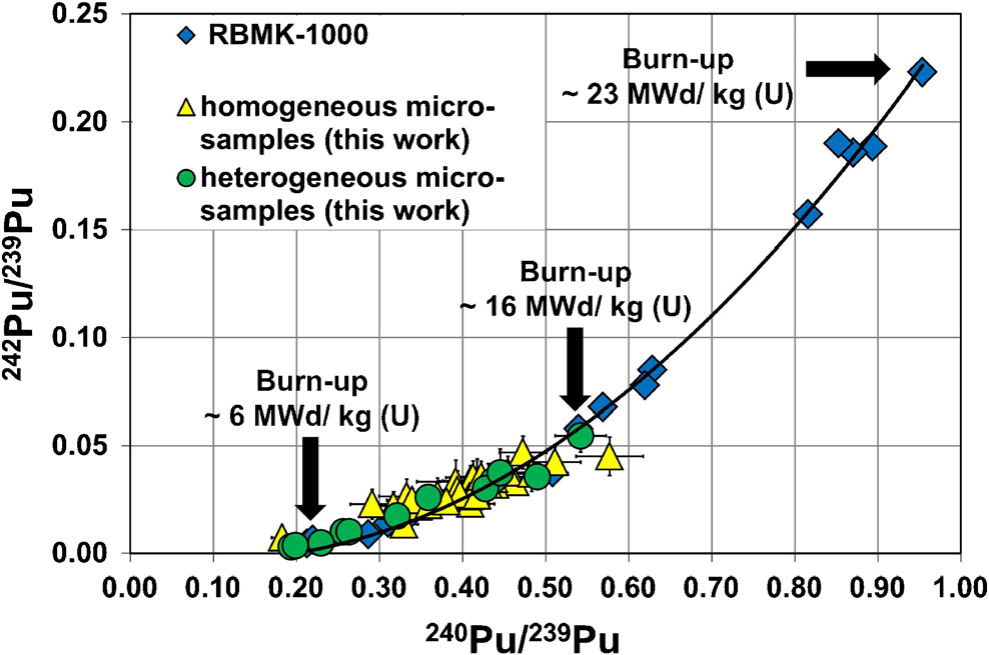
Comparison of the Pu isotopic distribution measured in micro-samples from the Chernobyl fallout with a typical Pu isotopic distribution of the RBMK-1000 reactor [48] having an initial ^235^U enrichment of ∼2 %

In Fig. 6, the measured Pu isotopic signatures are compared with the Pu isotopic composition distribution that is typical for a RBMK-1000 reactor type with an initial ^235^U enrichment of about 2 %. The latter data, which were published by the Oak Ridge National Laboratory [48], can be taken for comparison in the first approximation as the RBMK-1000 Chernobyl reactor exhibited similar characteristics. The Pu isotopic distribution, which is plotted according to increasing burn-ups of the spent fuel of the RBMK-1000 reactor, follows a polynomial curve. As can be seen from Fig. 6, such a curve does also fit the distribution of the Pu isotopic signatures of all micro-samples (*n* = 48) analyzed in this study, except for a few samples, which slightly deviate from the predicted distribution. It is believed that these deviations are either resulting from mixed isotope ratios as a result of insufficient spatial resolutions of both nuclear track radiography and laser ablation or from specific peculiarities of the nuclear core parameters of the Chernobyl reactor, which were not accounted for in the data from the Oak Ridge National Laboratory [48].

## Conclusions

Overall, the fact that the determined Pu isotope ratios were in good agreement with the expected Pu isotopic composition over the Chernobyl reactor core demonstrates well that the combination of MC-ICP-MS and laser ablation represents a very powerful analytical method for determining accurate, spatially resolved actinide isotopic signatures in environmental samples. Its application for analyzing radioactive fuel particle clusters from the Chernobyl fallout allowed to gain new insights into the burn-up distribution of the reactor core at the time of the accident. Destruction-free particle pre-selection by means of nuclear track radiography has proven an important pre-requisite for saving valuable LA-MC-ICP-MS analysis time.

Moreover, this study demonstrates well that isotopic signatures can serve as strong indicators for linking material to their sources. However, it should be noted that the isotopic signature is only one indicator amongst many. Therefore, if available, more source attribution parameters (e.g. elemental impurities) should be taken into account for an unambiguous source attribution.
